# Expanding the limits of vaginal surgery: Transvaginal vesicolithotomy for an incarcerated procidentia: A case report and literature review

**DOI:** 10.1016/j.crwh.2024.e00624

**Published:** 2024-05-31

**Authors:** Themistoklis Mikos, Nikolaos Roussos, Iakovos Theodoulidis, Grigoris F. Grimbizis

**Affiliations:** 1^st^ Department of Obstetrics & Gynecology, Aristotle University of Thessaloniki, Papageorgiou General Hospital, Thessaloniki, Greece

**Keywords:** Bladder stone, Pelvic organ prolapse, Incarcerated procidentia, Transvaginal vesicolithotomy, Case report

## Abstract

Bladder stones are rare in women. This report presents the case of a woman with a massive bladder stone and incarcerated procidentia. The 75-year-old woman presented to the outpatient clinic with procidentia and recurrent urinary tract infections. Preoperative imaging led to the diagnosis of cystolithiasis. After multidisciplinary counseling the patient underwent a vaginal hysterectomy with bilateral oophorectomy and transvaginal vesicolithotomy. A bladder biopsy was performed to rule out any malignancy. After three days, the patient was discharged with a Foley catheter; 15 days later, the bladder catheter was removed. She had an uncomplicated postoperative course. The presence of cystolithiasis and pelvic organ prolapse remains a challenge both in diagnosis and in treatment. The literature lacks solid evidence on the optimal management of these cases. Although there are no recommendations or consensus for their treatment, it seems that the one-step vaginal approach is preferable to the abdominal route.

## Highlights

• Pelvic organ prolapse may be related to bladder stone formation.
• Uterine incarcerated procidentia may be caused by a bladder stone.
• Vesicolithotomy is feasible *via* the vaginal route with minimal complications.
• One-step transvaginal vesicolithotomy combined with prolapse surgery is adopted in most of these cases.

## 1 Introduction

Female urinary stone formation is uncommon. Outlet obstruction and foreign materials are the main causes of calculus formation in women with non-neurogenic bladder disease. Pelvic organ prolapse (POP) represents the main cause of bladder outlet obstruction in women [1]. There is some evidence in the literature linking POP with vesical calculi formation.

A small number of patients with incarcerated procidentia and bladder calculus are reported in the literature [1]. The present report describes a rare case of persistent multi-compartment genital prolapse that caused bladder outlet blockage co-existing with a massive bladder stone. The combination of these two pathologies probably had as a result an incarcerated procidentia that was surgically treated using a comprehensive combined transvaginal method. Furthermore, a thorough review of the literature was conducted in order to identify any association between POP and vesical calculi formation and to elucidate the most appropriate treatment.

## 2 Case Presentation

A 75-year-old Caucasian woman (para 3, vaginal deliveries 3, BMI 31, not sexually active) presented to the outpatient clinic with non-reducible prolapse, pain, voiding difficulties, frequency, urgency and urge urinary incontinence. Moreover, she reported recurrent urinary tract infections, for which she had been offered multiple medical treatments. The patient had a medical history of diabetes, hypertension and coronary artery disease. She reported procidentia over 7 years; the symptoms, however, had deteriorated over the previous 3 months.

Upon admission, the patient consented to potential publication of the relevant clinical information. The physical examination showed an incarcerated uterine procidentia with mucosal ulcerations (POP-Q: Aa = +3, Ba = +11, C = + 13, GH = 4, PB = 3, TVL = 13, Ba = +3, Bp = +11, D = +11). Stress cough test was negative; occult stress urinary incontinence could not be checked as the prolapse could not be reduced without sedation. Ultrasound examination and computerized tomography (CT) revealed a small uterus (4 × 2.5 cm), a long cervix (6 cm in length), post-void residual volume (PVR) of 180 ml, and a bladder calculus with 8 cm maximum diameter.

**Fig. 1:**
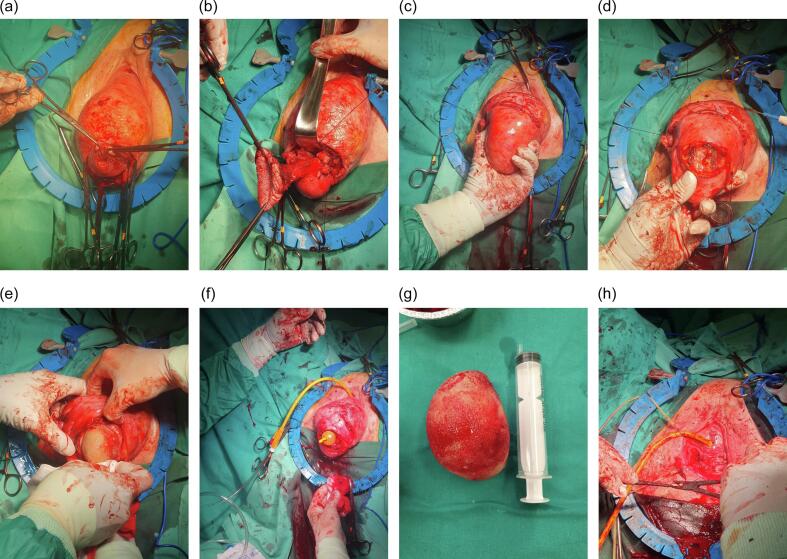
Transvaginal transperitoneal vesicolithotomy of an 8 cm bladder stone in a patient with incarcerated uterine procidentia. (a) Beginning of vaginal hysterectomy: a circumcised incision around the uterine cervix. (b) Advanced steps of vaginal hysterectomy: ligation of the ovarian pedicles. (c) After removal of the uterus: the bladder with the stone is immobilized in the surgeon's left hand. (d) Vesicolithotomy – 1: An initial transverse incision on the perineum of the bladder and the bladder wall. (e) Vesicolithotomy – 2: the bladder is opened, and the surgeon attempts to ‘deliver’ the stone. (f) Vesicolithotomy – 3: The stone has been removed and the Foley catheter is left hanging through the bladder incision. (g) An 8-cm bladder stone. (h) Completion of the prolapse surgery with total colpectomy and perineorrhaphy.

After extensive counseling on treatment options, the patient chose the less invasive surgical treatment. After discussing the case in a multidisciplinary meeting (gynecologist, urologist, anesthetist, cardiologist) a trial of vaginal hysterectomy, removal of the bladder stone, and then colpocleisis was decided upon. After she gave informed consent, the patient was placed in the lithotomy position. Under spinal anesthesia, vaginal hysterectomy with bilateral salpingo-oophorectomy was initially performed (Fig. 1a – 1c). As the prolapse remained non-reducible, a transvaginal transperitoneal cystolithotomy was performed (Fig. 1d – 1h). First, the bladder peritoneum was reversed, then a transverse incision on the peritoneum was performed and a longitudinal incision through the detrusor layers was made to permit the 8 cm renal calculus to be manually extracted. A bladder biopsy was performed to rule out any malignancy. The bladder wall, which was watertight, was repaired in two layers with Polyglactin 910 3.0 sutures. A third layer of continuous Polyglactin 910 2.0 was used to close the bladder peritoneum. A cyanide blue test (180 ml) was conducted and yielded a negative result. Peritoneal closure of the initial colpotomy was performed subsequently. Finally, a total colpectomy with purse-string sutures and a perineorrhaphy concluded the reconstruction. Blood loss during the 120-min operation was estimated to be 300 ml. After three days, the patient was discharged with normal renal parameters; 3 months later, the patient was doing well, with no incontinence and no prolapse recurrence. She was satisfied but chose to make no comment regarding her perspectives on the treatment she had received for the purposes of publication.

## 3 Discussion

Cystolithiasis or bladder stones refer to the presence of materials consisting of calcium, uric acid, oxalate and phosphate in the urinary bladder. They primarily affect men over 50 years old due to benign prostatic hyperplasia that causes bladder outlet obstruction. Nevertheless, women presenting with pelvic organ prolapse can also be affected [2]. Despite being two different entities, some researchers suggest a possible connection between POP and vesicolithiasis. POP can be a risk factor for bladder stones due to bladder outlet obstruction and urinary stasis that predisposes to urinary tract infections contributing to the urea breakdown [2]. Urinary stasis due to POP appears to be the primary risk factor that contributed to the formation of stones in the case reported here.

Patients with cystolithiasis mainly complain about pain and voiding difficulties. In most cases the detection of bladder stones is incidental, *via* imaging techniques prior to surgery. When non-reducible procidentia is accompanied by chronic pain and voiding problems, the possibility of bladder calculi should be considered. In these women, the surgical intervention should be considered urgent, and the gynecologist should contemplate preoperative urinary tract imaging to mitigate the risk of overlooking bladder calculi. In the present case, a CT scan detected the urolithiasis. Bladder stones can also be detected with X-rays or ultrasound. However, CT is the gold standard examination for the diagnosis of urolithiasis. In case of cystolithiasis, bladder biopsy is necessary because of the high association between bladder calculi >25 mm and malignancy [1].

The surgical management of POP and urolithiasis remains controversial. The literature does not provide clear answers as to whether the prolapse procedure and vesicolithotomy should be performed simultaneously or in two steps, which procedure should be prioritized, and which route is the most appropriate.

A thorough literature search was conducted using the PubMed database to identify case reports of simultaneous advanced pelvic organ prolapse and cystolithiasis. The keywords were ‘pelvic organ prolapse’, ‘POP’, ‘procidentia’, ‘cystolithiasis’, ‘bladder calculus’, and ‘bladder stone’. The search was restricted to studies that were published through to December 2023. The title and abstract of each article were screened to identify their relevance. Subsequently, relevant articles published in English were retrieved and reviewed.

The literature review identified 27 articles reporting 28 cases of women with POP and vesicolithiasis (Table 1). The mean age was 69.3 ± 8.1 years, and the mean duration of symptoms was 8.2 ± 9.1 years. Uterine prolapse was the commonest type of POP (89.6%), and the mean number and size of the bladder calculi was 14.9 ± 20.2 and 5.3 ± 2.4, respectively. Hysterectomy was performed in 65.4% (17/26) of the cases, and a concomitant anti-incontinence procedure in 5 cases [Burch colposuspension in 4 (22.3%), and retropubic TVT in 1 (5.5%)]. Mean postoperative follow-up was 6.3 ± 6.7 months.

**Table 1:** Published cases of incarcerated procidentia and bladder calculi.

Author, Year	Age (Years)	Duration (years)	POP	Calculi		Cystotomy	Hysterectomy	POP Sx	SUI Sx	Follow-up (months)	Comments
				Number	Max Size (cm)						
**Suprapubic Approach**											
Mann, 1958 [17]	62	5	Uterus	20	3.2	Suprapubic	No	TV repair, VH	No	26	2-step procedure
	71	23	Uterus	13	4.5	Suprapubic	No	TV repair, VH	No	7	2-step procedure
Nieder, 1998 [18]	82	>1	Vault	>15	N/A	Suprapubic	No	TV repair	No	2	None
Dalela, 1999 [19]	70	N/A	Uterus	1	5	Suprapubic	No	TV repair, VH	No	N/A	None
Wai, 2003 [20]	72	>2	Vault	>15	2.5	Suprapubic	No	Colpocleisis	Burch	12	None
Siriwardana, 2008 [21]	60	10	Uterus	4	N/A	Suprapubic	Yes	TV repair, VH	No	12	None
Rajamaheswari, 2012 [22]	57	20	Uterus	>100	10	Suprapubic	Yes	TV Repair	No	4	Post-op ileus
Thompson, 2018 [24]	71	7	Uterus	>30	5	Suprapubic	Yes	Abdo/Vaginal	Burch	3	Wound Infection, Rectus Fascia dehiscence
Garg, 2019 [23]	65	10	Uterus	>30	N/A	Suprapubic	Yes	TV repair, VH	No	3	None
Chaudhari et al., 2022 [25]	60	N/A	Uterus, Rectus	1	N/A	Suprapubic	Yes	TAH, SCP, Rectopexy, TV repair	Burch	N/A	None
Komon et al., 2023 [26]	77	10	Uterus	1	6	Suprapubic	Yes	TAH, SCP	No	3	None
**Subtotal**	68	10		20	5.2					8	
											
**Vaginal Approach**											
Johnson, 1958 [8]	54	3	Uterus	1	8	Vaginal	Yes	Colpectomy	No	6	None
Svesko, 1958 [9]	81	1	Uterus	1	4	Vaginal	No	Colpectomy		3	None
Mahran, 1972 [10]	84	35	Uterus	9	8	Vaginal	No	No	No	3	No POP Sx
Chambers, 1975 [3]	76	3	Uterus	>15	2.6	Vaginal	No	TV repair, VH	No	N/A	2-step procedure
Pranikoff, 1982 [4]	74	N/A	Uterus	>15	8	Vaginal	No	No		18	Vasicovaginal Fistula Pessary, No POP Sx
Partoll, 1996 [7]	74	3	Uterus	1	6.5	Vaginal	Yes	Abdo/Vaginal	Burch	1	Pneumonia
Megadhana, 2006 [11]	76	9	Uterus	3	5	Vaginal	Yes	Colpocleisis	No	2	None
Agarwal, 2007 [12]	70	3	Uterus	1	8.5	Vaginal	Yes	TV repair, VH	No	1.5	None
Dahiya, 2007 [13]	60	15	Uterus	50	N/A	Vaginal	Yes	TV repair, VH	No	1 m	None
Washington, 2008 [14]	71	3	Uterus	>15	8.1	Vaginal	Yes	SSLF	No	N/A	None
Naidu, 2011 [15]	76	N/A	Uterus	>30	2.5	Vaginal	Yes	Colpocleisis	No	6	Uterine Inversion
Hudson, 2014 [16]	76	22	Uterus	10	2	Vaginal	No	Colpocleisis	No	3	None
AlSary et al., 2023 [6]	56	12y	Uterus	>30	4 cm	Vaginal	Yes	TV repair, VH, SSLF	No	N/A	Initial pessary & Local E_2_
**Subtotal**	72	8		13	6					4	
											
**Lithotripsy**											
Kang, 2000 [27]	66	6	Uterus	2	N/A	Lithotripsy	No	TV repair, VH	No	18	Acute Renal Failure 2-step procedure
Hiremath, 2019 [29]	65	10	Vault	6	2.8	Cysto-Litholapaxy	No	SCP	No	N/A	None
Ramalingam et al., 2023 [28]	62	N/A	Uterus	1	2.7	Lithotripsy	Yes	N/A	N/A	N/A	2-step procedure
**Subtotal**	64	5		3	3					18	
											
**Total**	69.3 ± 8.2	8.2 ± 9.1		14 ± 20	5.3 ± 2.4					6.3 ± 6.7	

POP=Pelvic Organ Prolapse, Sx = Surgery, SUI=Stress Urinary Incontinence, TV = TransVaginal, VH=Vaginal Hysterectomy, TAH = Total Abdominal Hysterectomy, SCP=SacroColpoPexy, SSLF=SacroSpinous Ligament Fixation,

Three main approaches for surgical removal of the bladder stone have been reported in the literature: the transvaginal approach (14/29, 48.3%) [[3], [4], [5], [6], [7], [8], [9], [10], [11], [12], [13], [14], [15], [16]], the suprapubic approach (11/29, 37.9%) [[17], [18], [19], [20], [21], [22], [23], [24], [25], [26]], and lithotripsy (3/29, 10.3%) [[27], [28], [29]]; there is also a report of spontaneous passing of a 3 cm stone after vaginal packing of POP [5]. The main advantages of the transvaginal approach appear to be the reduced surgical trauma in this elderly, rather frail population, the use of local rather than general anesthesia, less peri-operative bleeding, less postoperative pain, and quick mobilization of the patient. This approach seems to be suitable even for the large bladder calculi, measuring up to 8.5 cm [12]. It can be combined with vaginal hysterectomy and salpingo-oophorectomy, as in the present case. In the present case, a transperitoneal incision was decided upon, in order to minimize the possibility of postoperative vesicovaginal fistula; this was greatly facilitated, however, by the complete inversion of the pelvic organ prolapse and the bladder wall. In terms of complications, a case of pneumonia and a case of vesicovaginal fistula have been reported after vaginal vesicolithotomy and combined prolapse surgery [4,7].

The suprapubic approach appears to offer the advantages of better surgical access to the bladder and the ureters; it also gives the opportunity of intra-operative insertion of ureteric stents, and it avoids simultaneous neighboring incisions to the bladder and the vagina, a factor favoring postoperative vesicovaginal fistula formation. In the literature, the suprapubic approach has been accompanied by vaginal hysterectomy (5/11, 45.5%), abdominal hysterectomy (3/11, 27.3%), abdominal sacrocolpopexy (2/11, 18.2%), transvaginal prolapse repair (9/11, 81.8%), and Burch colposuspension (3/11, 27.3%). These procedures appeared to have a low complication rate: a case of postoperative ileus [22], and a case of wound infection and rectus fascia dehiscence for which the patient was required placement of a mesh [24].

Lithotripsy appears to have the advantages of being a minimally invasive non-surgical procedure, as well the avoidance of the one of the necessary interventions (surgical stone removal *and* POP repair); the patient benefits from the possibility of insertion of ureteric stents as well. Three patients are reported in the literature who were treated with lithotripsy and POP surgery: one had a subsequent vaginal hysterectomy, one had a sacrocolpopexy, and the third had abdominal hysterectomy. One of these patients had acute renal failure. Lithotripsy appears to be a reasonable option when adopting a two-step procedure, was it means the woman does not have to undergo two major interventions.

Most cases reported in the literature suggest the simultaneous management of the POP and the bladder stone (a one-step procedure, in 24 cases, 82.7%) (Table 1). From the 14 patients who had a vaginal approach, only one had a two-step procedure: the bladder stone was initially removed and 52 days later a vaginal hysterectomy with anterior-posterior repair was performed [3]. However, Mann *et al* first reported the two-step approach, and described the management of urolithiasis with suprapubic cystolithotomy and after 12 days performed a vaginal hysterectomy [17]. In 2000, Kang *et al* in their case report first removed the bladder stones with lithotripsy and 8 days later performed a vaginal hysterectomy [27]. Ramalingam *et al* first performed lithotripsy and after 2 days a hysterectomy [28]. Despite the lack of sufficient data, in terms of complications it seems that the two-step procedure is comparable to the simultaneous management. The removal of the bladder stones should be the first step in these cases, permitting the alleviation of symptoms, the feasibility of POP reduction, the reduction of inflammation, and providing adequate surgical space for a vaginal approach. Apparently, the two-step approach may be offered in centres that cannot offer surgical management of POP. Nevertheless, the optimal interval between the two procedures is yet to be determined.

It is crucial to emphasize that the published case reports feature a cohort of women with bladder stones that differ in size and quantity. The latter can be crucial factors, altering the management of the urolithiasis. Additionally, the surgical experience of the operative team may influence the choice of surgical technique. Furthermore, the follow-up period documented in the literature varies widely.

## 4 Conclusion

Managing POP with cystolithiasis is challenging and may necessitate a multidisciplinary approach. Cystolithiasis, albeit uncommon, should be taken into account when encountering the issue of incarcerated procidentia. The surgical intervention should be considered urgent, and the vaginal approach for cystolithotomy seems preferable to the abdominal approach, but caution must be exercised to prevent any potential harm to the ureter and the formation of a fistula.
